# Comparison between sevoflurane and propofol on immunomodulation in an *in vitro* model of sepsis

**DOI:** 10.3389/fmed.2023.1225179

**Published:** 2023-07-27

**Authors:** Tainá B. Oliveira, Cassia L. Braga, Denise Battaglini, Paolo Pelosi, Patricia R. M. Rocco, Pedro L. Silva, Fernanda F. Cruz

**Affiliations:** ^1^Laboratory of Pulmonary Investigation, Institute of Biophysics Carlos Chagas Filho, Federal University of Rio de Janeiro, Rio de Janeiro, Brazil; ^2^Anesthesia and Critical Care, San Martino Policlinico Hospital, IRCCS for Oncology and Neurosciences, University of Genoa, Genoa, Italy; ^3^Department of Surgical Sciences and Integrated Diagnostics, University of Genoa, Genoa, Italy

**Keywords:** sepsis, anesthetics, sevoflurane, propofol, immunomodulation, anti-inflammatory

## Abstract

**Introduction:**

Patients with sepsis often require sedation and/or anesthesia. Although the immunomodulatory effects of anesthetics have been increasingly recognized, the molecular mechanisms require better elucidation. We compared the effects of sevoflurane with propofol on the expression of pro- and anti-inflammatory biomarkers released by monocytes/macrophages and blood/bronchoalveolar lavage fluid (BALF) neutrophils, the phagocytic capacity of monocytes/ macrophages, and neutrophil migration, as well as mediators associated with alveolar epithelial and endothelial cells obtained from rats with sepsis.

**Methods:**

Polymicrobial sepsis was induced by cecal ligation and puncture in nine male Wistar rats. After 48 h, animals were euthanized and their monocytes/alveolar macrophages, blood and BALF neutrophils, as well as alveolar epithelial and endothelial cells were extracted, and then exposed to (1) sevoflurane (1 minimal alveolar concentration), (2) propofol (50 μM), or (3) saline, control (CTRL) for 1 h.

**Results:**

Sevoflurane reduced interleukin (IL)-6 mRNA expression in monocytes and alveolar macrophages (*p* = 0.007, *p* = 0.029), whereas propofol decreased IL-6 mRNA only in alveolar macrophages (*p* = 0.027) compared with CTRL. Sevoflurane increased IL-10 expression (*p* = 0.0002) in monocytes compared with propofol and increased IL-10 mRNA and transforming growth factor (TGF)-β mRNA (*p* = 0.037, *p* = 0.045) compared with CTRL. Both sevoflurane and propofol did not affect mRNA expression of IL-10 and TGF-β in alveolar macrophages. The phagocytic capacity of monocytes (*p* = 0.0006) and alveolar macrophages (*p* = 0.0004) was higher with sevoflurane compared with propofol. Sevoflurane, compared with CTRL, reduced IL-1β mRNA (*p* = 0.003, *p* = 0.009) and C-X-C chemokine receptor 2 mRNA (CXCR2, *p* = 0.032 and *p* = 0.042) in blood and BALF neutrophils, and increased CXCR4 mRNA only in BALF neutrophils (*p* = 0.004). Sevoflurane increased blood neutrophil migration (*p* = 0.015) compared with propofol. Both sevoflurane and propofol increased zonula occludens-1 mRNA (*p* = 0.046, *p* = 0.003) in alveolar epithelial cells and reduced Toll-like receptor 4 mRNA (*p* = 0.043, *p* = 0.006) in alveolar endothelial cells compared with CTRL. Only propofol reduced surfactant protein B mRNA (*p* = 0.028) in alveolar epithelial cells.

**Discussion:**

Sevoflurane, compared with propofol, increased anti-inflammatory biomarkers in monocytes, but not in alveolar macrophages, enhanced monocyte/alveolar macrophage phagocytic capacity and increased neutrophil migration in *in vitro* experimental sepsis. Both propofol and sevoflurane protected lung epithelial and endothelial cells.

**Graphical Abstract:**
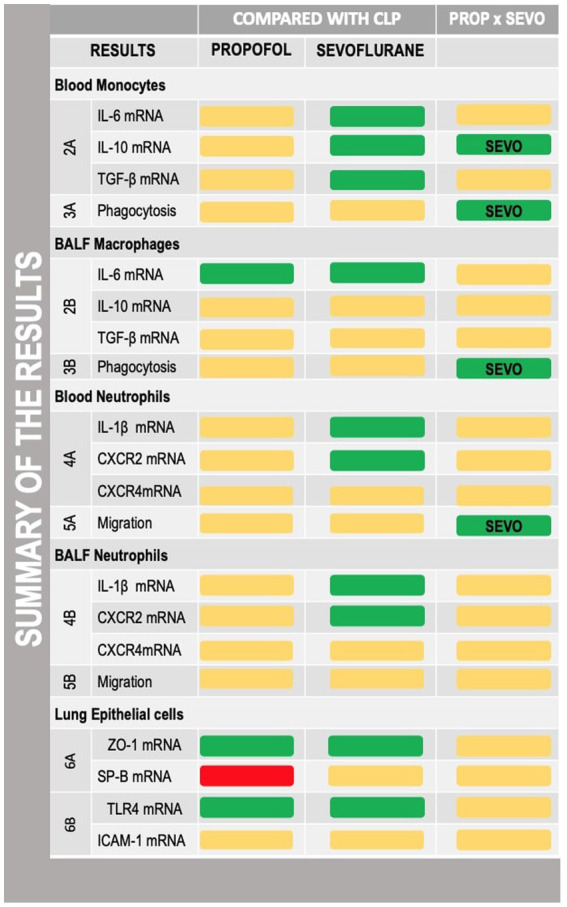
Summary of the results described in the manuscript with reference to the figures where the results were described. CLP, cecum ligation and puncture, control group, not exposed to anesthetics; MAC, minimum alveolar concentration; PROP, propofol group, exposed to 50 μM propofol; SEVO, sevoflurane group, exposed to sevoflurane 1 MAC; IL, interleukin; TGF-b, transforming growth factor beta; CXCR, C-X-C motif chemokine receptor; SP-B, surfactant protein B; TLR4, Toll-like receptor 4; ZO-1, zona occludens 1. Yellow box, no difference; green box, protective; red box, deleterious.

## 1. Introduction

Sepsis is a health care burden with high morbidity and mortality (1, 2). General anesthesia is administered to patients with sepsis for surgical and diagnostic procedures and for sedation in critically ill patients admitted to the intensive care unit (ICU) (1, 3, 4).

Propofol is the most widely used intravenous anesthetic for induction of short-term anesthesia (5). Studies have demonstrated differing actions of propofol in sepsis: reduction in neutrophils and suppression of Toll-like receptor (TLR) 4-mediated inflammation by inhibiting activation of nuclear factor (NF)-κB (6) or increased pro-inflammatory mediators, morbidity, and mortality (7).

Sevoflurane is a commonly used volatile anesthetic agent for general anesthesia during surgical interventions. It is characterized rapid induction and recovery (8), low airway irritability, and adequate hemodynamic properties (9). The use of volatile anesthetics for sedation of critically ill patients in ICUs has become feasible (10–13) with the advent of devices that allow adaptations in the ventilator circuit; for example, mini-vaporizers (MIRUS, TIM, Koblenz, Germany or AnaConDa, Sedana Medical, Stockholm, Sweden). The use of volatile anesthetics in ICUs increased during the coronavirus 2019 pandemic due to shortages of venous anesthetic agents (11). In critically ill patients, the administration of sevoflurane decreased wake-up and extubation times, morphine consumption post extubation, and increased awakening quality compared with propofol (14). In experimental sepsis, sevoflurane modulates the inflammatory process (15), improves survival (16), decreases NF-κB translocation into monocytes (17), and enhances macrophage phagocytosis (18).

Even though many mechanisms of action concerning the effects of propofol and sevoflurane in sepsis have previously been described, the effects of these anesthetic agents on specific inflammatory cells (circulating monocytes, neutrophils and bronchoalveolar lavage fluid macrophages) as well as on lung structural cells (lung epithelial and endothelial) require elucidation. In the clinical setting, it is difficult to isolate the effects of anesthetic agents from those of surgical stress or other individual covariates. Thus, *in vitro* (19) analysis of immune and structural cells from septic animals may help to better elucidate the molecular mechanisms of anesthetics.

We hypothesized that the exposure of inflammatory and lung structural cells to sevoflurane compared with propofol led to less inflammation and greater phagocytic capacity in sepsis. Our primary outcome was the impact of anesthetics on the mRNA expression of the pro-inflammatory mediator, interleukin (IL)-6. Secondary outcomes included the effects of sevoflurane and propofol on the expression of other pro-and anti-inflammatory biomarkers released by monocytes/alveolar macrophages and blood/bronchoalveolar lavage fluid (BALF) neutrophils, the phagocytic capacity of monocytes/macrophages, and neutrophil migration, as well as mediators associated with lung epithelial and endothelial cells obtained from septic rats.

## 2. Materials and methods

### 2.1. Experimental conditions

This study was approved by the Institutional Animal Care and Use Committee of the Health Sciences Centre, Federal University of Rio de Janeiro (CEUA 027/17). All animals received humane care in compliance with the Principles of Laboratory Animal Care proposed by the National Society for Medical Research and the US National Academy of Sciences *Guide for the Care and Use of Laboratory Animals*. This study followed the ARRIVE guidelines for reporting of animal research (20). Conventional animals were housed at a controlled temperature (23°C) in a controlled light–dark cycle (12–12 h), with free access to water and food.

### 2.2. Experimental groups and timeline

**Figure 1:**
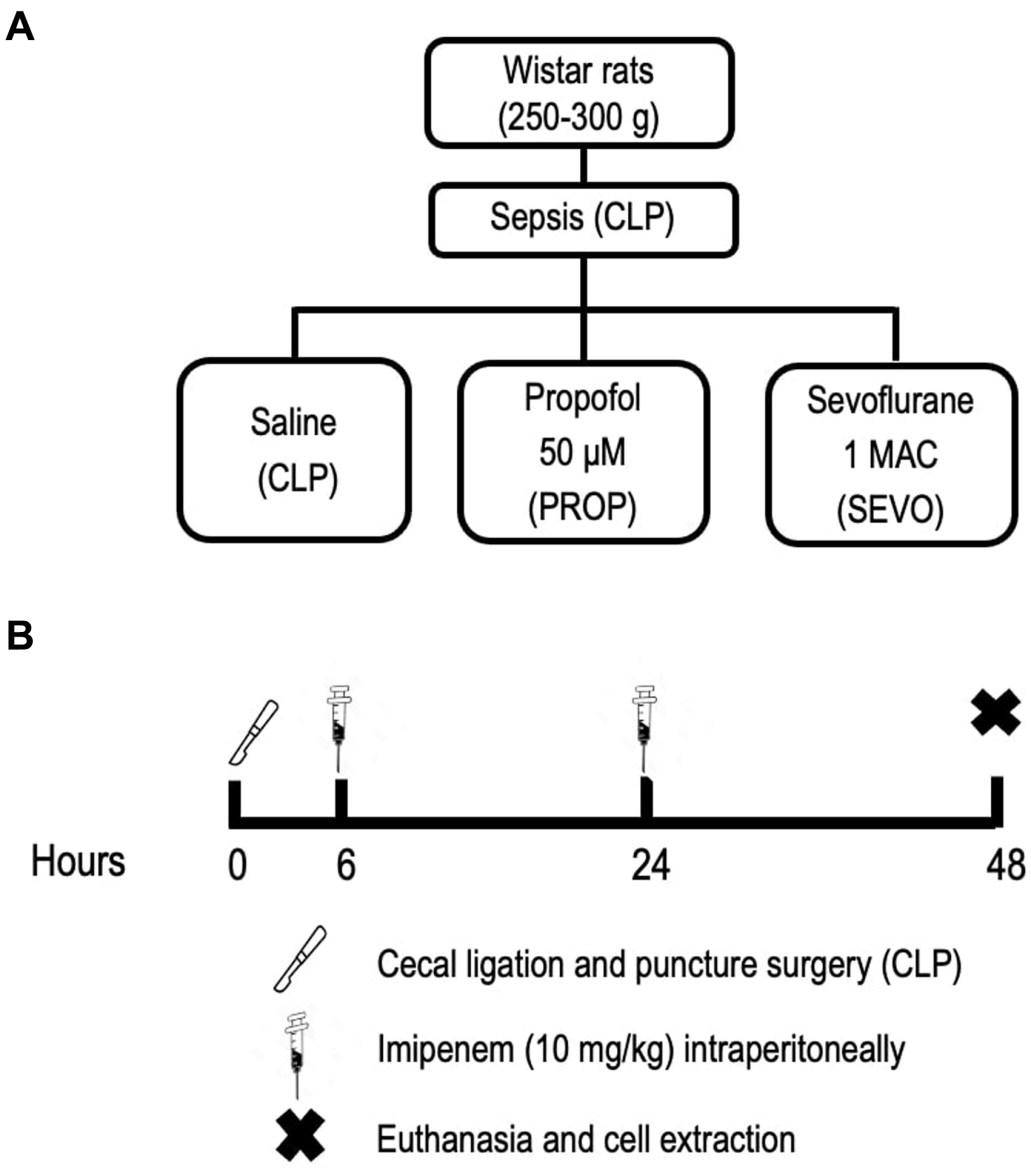
Experimental design. **(A)** Experimental groups. **(B)** Experimental timeline. CLP, cecum ligation and puncture, control group, not exposed to anesthetics; MAC, minimum alveolar concentration; PROP, propofol group, exposed to 50 μM propofol; SEVO, sevoflurane group, exposed to sevoflurane, 1 MAC.

Nine male Wistar rats (290 ± 20 g; 6 ± 1 weeks old; Figure 1A) were obtained from the Health Science Center, Federal University of Rio de Janeiro. Polymicrobial sepsis was induced by cecal ligation and puncture (CLP), as described previously (21). Briefly, animals were anesthetized with intraperitoneal (ip) injection of midazolam (5 mg/kg body weight) and ketamine (60 mg/kg body weight) (Cristália, São Paulo, Brazil). After anesthesia and local disinfection, a small 2-cm incision was made in the lower portion of the abdomen. A longitudinal incision was made through the skin, muscular, fascial, and peritoneal layers. The cecum was exposed and approximately 25% of its area was ligated, avoiding obstruction so that intestinal transit was not interrupted. The bound area of the cecum was then perforated twice with an 18G needle, followed by digital compression for stool leakage through the puncture holes into the peritoneal cavity. The intestinal segment was reinserted into the abdominal cavity, followed by flat closure of both abdominal layers using nylon 3.0 suture. All animals received resuscitation fluid after surgery (5 mL of saline heated at 37°C/100 g body weight, subcutaneously) to better simulate the initial hyperdynamic phase of sepsis. Tramadol (0.05 mg/kg body weight subcutaneously) was used for postoperative analgesia, every 8 h until the end of the protocol. After recovery from anesthesia, the animals were returned to their cages. Six and 24 h after sepsis induction, the rats received the antibiotic imipenem (10 mg/kg body weight, ip) in the opposite site from the surgery (22). Forty-eight hours after induction of sepsis, the animals were sedated with diazepam (4 mg/kg body weight, ip) followed by euthanasia with a lethal dose of thiopental sodium (150 mg/kg body weight, ip) (Figure 1B). Additionally, we have used a clinical score to assess animal’s well-being. If animals presented score indicating suffering before ending protocol, they were euthanized and not used for this study (23). BALF and blood were collected to isolate neutrophils and macrophages/monocytes. Lungs were removed and pulmonary endothelial and alveolar epithelial cells were primarily extracted, following specific protocols (24). Cells were then exposed either to saline (negative control group, CLP), propofol (PROP), or volatile anesthetic sevoflurane (SEVO) for 1 h (Figure 1A).

### 2.3. Bronchoalveolar lavage fluid, blood, and lung extraction

Tracheostomy was performed aseptically, and an 18G cannula was inserted and attached to a syringe containing 3 mL of sterile preheated 1× phosphate buffered solution (PBS). Bronchoalveolar lavage was repeated three times. Blood was obtained by aspiration of the inferior vena cava with a heparinized syringe. The lungs and heart were removed immediately *en bloc* and immersed in 1× PBS until processed.

#### 2.3.1. Isolation of neutrophils and macrophages from BALF and blood

BALF was centrifuged (300×*g*, 5 min) and the cell pellet was resuspended in 2 mL od 1× PBS. Blood and BALF cells were slowly transferred to a tube containing Ficoll-Paque (Sigma, St Louis, MO, United States), then centrifuged (400×*g* at 20°C) for 30 min to isolate mononuclear and polymorphonuclear cells over the density gradient created by centrifugation (25–29). Mononuclear and polymorphonuclear cells were then treated with red blood cell lysis buffer (Sigma) for 5 min, washed twice with 1× PBS, and centrifuged (400×*g* at 20°C) for 5 min. Cells were then resuspended in Roswell Park Memorial Institute culture medium (RPMI) supplemented with fetal bovine serum (3% FBS) and 1% penicillin/streptomycin (penicillin 100 U/mL and streptomycin 100 U/mL; Invitrogen, Life Technologies, Grand Isle, NY, United States). Cell viability was assessed by Trypan blue (above 90%), and 10^5^ neutrophils or macrophages/monocytes were immediately plated in petri dishes (60 × 15 mm; Eppendorf, Hamburg, Germany) to be randomly divided into the three exposure groups.

#### 2.3.2. Isolation of pulmonary endothelial cells and alveolar epithelial cells

##### 2.3.2.1. Pulmonary endothelial cells

The left lung was isolated, cut with scalpel blades, and transferred to a polystyrene tube with 2 mL of 1% collagenase solution (Sigma) in a water bath at 37°C under agitation for 40 min. Tissue digestion was stopped with Iscove’s medium (IMDM) containing 20% FBS and 1× penicillin/streptomycin, filtered over a 70-μm cell strainer, and centrifuged (300×*g*, 5 min). Cells were washed three times with 1× PBS, and endothelial cells were selected by magnetic sorting with biotinylated anti-PECAM antibody (BioLegend, San Diego, CA, United States, 1:1000), followed by Dynabeads (Thermo Fisher, Waltham, MA, United States) incubation and exposure to magnetic field. Endothelial cells were plated into 0.2% (w/v) sterile gelatin-coated petri dishes. Cells were kept in a humidified incubator (5% CO_2_, 37°C) with IMDM medium (Invitrogen, Life Technologies) supplemented with 20% calf serum (Hyclone, Rockford, IL, United States) and 1% penicillin/streptomycin, changed twice in 1 week, until 90% confluence (24, 30).

##### 2.3.2.2. Alveolar epithelial cells

The right lung and heart were removed for alveolar epithelial cell isolation as described previously (24, 30, 31). Briefly, 1X PBS with heparin (2 IU/mL) was perfused into the right ventricle to wash lung capillary blood. A tracheal cannula was used to slowly perfuse the lung three times with 1 mL of PBS Ca-Mg-free solution containing 0.2 mM egtazic acid. Connective tissues were removed, and the lungs were perfused with heated (37°C) Ham-F12 elastase solution (4 IU/mL) for 30 min, cut into small pieces, and incubated with heated (37°C) DNase I (50 mg/mL; Sigma). After 15 min, the cells were washed with Hank’s buffered saline solution (amphotericin, 250 μg/mL; 1 M HEPES, 0.5 M EDTA, 2% FBS, 1% penicillin/streptomycin), filtered over a 70-μm cell strainer, blocked by addition of bovine serum, and centrifuged (600×*g*, 4°C for 5 min). Cells were treated with red blood cell lysis buffer (Sigma), washed with 1× PBS, resuspended in culture medium supplemented with 10% FBS, and plated on 3 mg/mL collagen-coated petri dishes. Medium was changed twice a week until reaching 90% confluency (24, 30).

### 2.4. Cell exposure to anesthetics

Macrophages/monocytes and neutrophils from BALF and blood were exposed to anesthetic or saline as soon as cells were isolated. Pulmonary endothelial cells and alveolar epithelial cells were exposed to anesthetic or saline after adhering to the plates and reaching 90% confluency, 1 week after cell isolation. Cells were exposed to 1 minimum alveolar concentration (MAC, 2.0 vol%) sevoflurane (SEVO, Cristália, São Paulo, Brazil), 50 μM of commercial propofol (PROP, Cristália, São Paulo, Brazil) in serum free working cell media, which corresponds to the plasma concentration reached in clinical use (25–27, 32), or saline (CTRL) for 60 min. Sevoflurane was administered in a sealed acrylic chamber through a universal vaporizer (100 mL vaporizer; K. Takaoka, São Paulo, Brazil). The chamber also remained connected throughout exposure to the inhalant anesthetic to a multiparameter monitor (Networked Multiparameter Veterinary Monitor LifeWindow 6,000 V; Digicare Animal Health, Boynton Beach, FL, United States) for reliable measurement of sevoflurane and CO_2_ (5.0%) (15). Propofol (50 μM) was added to the same sealed acrylic chamber while controlling CO_2_ at 5%. After 60 min of exposure to anesthetic or saline, cells were harvested for chemotaxis and phagocytosis assays and gene expression by real-time reverse transcription polymerase chain reaction (RT-PCR).

### 2.5. Chemotaxis assay

After exposure to anesthetic or not, the migration of neutrophils was evaluated from an upper chamber through a 3-μm pore membrane into a lower chamber containing recombinant rat interleukin (IL)-8 (100 ng/mL) (33). The temperature in the chamber was controlled at 37°C, 5% CO_2_ for 1 h. Migration of neutrophils from the upper to the lower chamber was determined by the percentage of cells quantified in the bottom chamber.

### 2.6. Phagocytosis assay

The phagocytic capability of alveolar macrophages and blood monocytes was tested with pH-sensitive pHrodo Green *E. coli* BioParticles Conjugate for Phagocytosis (Invitrogen, Waltham, MA, United States), according to the manufacturer’s instructions. The conjugates are nonfluorescent at neutral pH, but fluoresce bright green at acidic pH, such as in phagosomes. Mononuclear cells collected from BALF or blood were seeded onto a tissue culture dish and incubated in RPMI 1640 10% fetal bovine serum (FBS) with 1% penicillin/streptomycin for 2 h at 37°C, 5% CO_2_. Cells that adhere to the plate are macrophages (26, 27, 34). Briefly, a total of 10^5^ alveolar macrophages were plated on a 96-well plate. Cells were washed with saline (0.9% NaCl) and incubated with fluorescent pHrodo Green *E. coli* BioParticles Conjugate for Phagocytosis (0.5 mg/mL) for 2 h. Phagocytosis was quantified by measuring intracellular fluorescence emitted by engulfed particles at 585 nm in a microplate fluorescence reader (PerkinElmer, Waltham, MA, United States).

### 2.7. Gene expression analysis by real-time RT-PCR

IL-10, IL-6, and transforming growth factor (TGF)-β mRNA expressions were measured in blood monocytes and alveolar macrophages. In blood and alveolar neutrophils, mRNA expressions of IL-1β (pro-inflammatory mediator) and neutrophil chemokine receptors (C-X-C motif chemokine receptor [CXCR]2 and CXCR4) were measured. Zonula occludens (ZO)-1 and surfactant protein (SP)-B were measured in alveolar epithelial cells. TLR4 and intercellular cell adhesion molecule (ICAM)-1 were measured in pulmonary endothelial cells. Total RNA was extracted with a ReliaPrep RNA Tissue Miniprep System (Promega, Fitchburg, WI, United States). The concentration of RNA was measured by spectrophotometry in a Nanodrop ND-2000 system. First-strand DNA was synthesized from total RNA using a High-Capacity cDNA Reverse Transcription Kit (Thermo Fisher). Relative mRNA levels were measured with the BRYT Green system (Promega) using a PCR Mastercycler ep Realplex system (Eppendorf). For each sample measured in duplicate, gene expression was normalized to that of a housekeeping gene (acidic ribosomal phosphoprotein P0, 36B4) and expressed as the fold change relative to the control group using the 2^−ΔΔCt^ method (35). The primers used are listed in Table 1.

**Table 1:** List of primers used.

Gene	Primer	Primer sequences (5′–3′)
Macrophages		
IL-6	Forward	CTC CGC AAG AGA CTT CCA G
	Reverse	CTC CTC TCC GGA CTT GTG A
IL-10	Forward	TCC CTG GGT GAG AAG CTG
	Reverse	GCT CCA CTG CCT TGC TCT
TGF-β	Forward	TAC AGG GCT TTC GCT TCA GT
	Reverse	TTG GTA TCC AGG GCT CTC C
Neutrophils		
IL-1β	Forward	CTA TGT CTT GCC CGT GGA G
	Reverse	CAT CAT CCC ACG AGT CAC A
CXCR2	Forward	CAA GCT GAT CAA GGA GAC CTG
	Reverse	CAA GGC CAT AAT TAG CCA TGA
CXCR4	Forward	GCA AGG ATG TGA GTT CGA GAG
	Reverse	CTC TGT GGA GAC GGA AGA GTG
Epithelium		
SP-B	Forward	CCA TCC CTC TGC CCT TCT G
	Reverse	CAC CCT TGG GAA TCA CAG CTT
ZO-1	Forward	CAC CAC AGA CAT CCA ACC AG
	Reverse	CAC CAA CCA CTC TCC CTT GT
Endothelium		
TLR4	Forward	CGG AAA GTT ATT GTG GTG T
	Reverse	GGA CAA TGA AGA TGA TGC CAG A
ICAM-1	Forward	CTT CCG ACT AGG GTC CTG AA
	Reverse	CTT CAG AGG CAG GAA ACA GG
Housekeeping		
36B4	Forward	AAT CCT GAG CGA TGT GCA G
	Reverse	GCT GCC ATT GTC AAA CAC C

IL, interleukin; TNF-α, tumor necrosis factor; TGF-β, transforming growth factor; CXCR, C-X-C motif chemokine receptor; SP-B, surfactant protein B; ZO-1, zona occludens 1; TLR4, Toll-like receptor 4; ICAM-1, intercellular adhesion molecule 1; 36B4, acidic ribosomal phosphoprotein P0, housekeeping gene.

### 2.8. Statistical analysis

A sample size of 9 animals provides the appropriate power (1 − β = 0.8) to identify significant (α = 0.05) differences in IL-6 gene expression in macrophages treated with sevoflurane for 1 h considering the size effect (*d* = 2.29), a two-sided test, and a sample size ratio of 1 (G*power 3.1.9.2; University of Dusseldorf, Dusseldorf, Germany). Data were tested for normality using the Kolmogorov–Smirnov test with Lilliefors’ correction, and the Levene median test was used to evaluate the homogeneity of variances. The Kruskal–Wallis test followed by Dunn’s multiple comparisons test was used for comparison of nonparametric data. Every group was compared with each other. Data are expressed as box and whisker plots. Boxes show the interquartile (25%–75%) range, whiskers encompass the range (minimum to maximum), and horizontal lines represent median values. All analyses were performed in a blinded manner (i.e., the observer was unaware of the experimental protocol) using the Prism version 8.1.1 software package (GraphPad Software, San Diego, CA, United States), and statistical significance was established at *p* < 0.05.

## 3. Results

**Figure 2:**
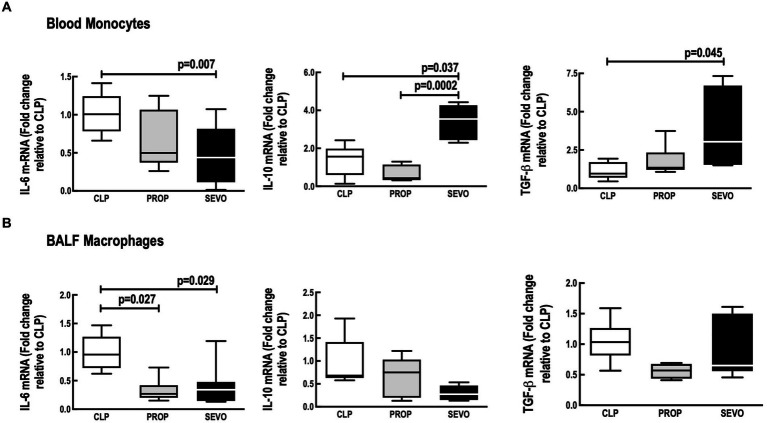
mRNA of mediators expressed by blood monocytes **(A)** and BALF macrophages **(B)**. BALF, bronchoalveolar lavage fluid; CLP, cecum ligation and puncture, control group, not exposed to anesthetics; MAC, minimum alveolar concentration; PROP, propofol group, exposed to 50 μM propofol; SEVO, sevoflurane group exposed to sevoflurane 1 MAC; IL, interleukin; TGF-β, transforming growth factor beta. Boxes show the interquartile (25%–75%) range, whiskers encompass the range (minimum to maximum), and horizontal lines represent median values of 9 animals/group.

Compared with CLP, Sevoflurane reduced IL-6 mRNA expression in blood monocytes (*p* = 0.007, Figure 2A) and alveolar macrophages (*p* = 0.029, Figure 2B), whereas propofol exposure reduced IL-6 mRNA expression only in BALF macrophages (*p* = 0.027, Figure 2B). No differences were observed between PROP and SEVO regarding IL-6 in blood monocytes and BALF macrophages.

In blood monocytes (Figure 2A), sevoflurane increased IL-10 mRNA expression (*p* = 0.037), and TGF-β (*p* = 0.045) compared with CLP, and increased IL-10 mRNA expression compared with propofol (*p* = 0.0002). No differences were observed between CLP versus PROP in blood monocytes IL-10 or TGF-β (Figure 2A). In BALF macrophages, the mRNA expression of IL-10 and TGF-β did not differ among the groups (Figure 2B).

**Figure 3:**
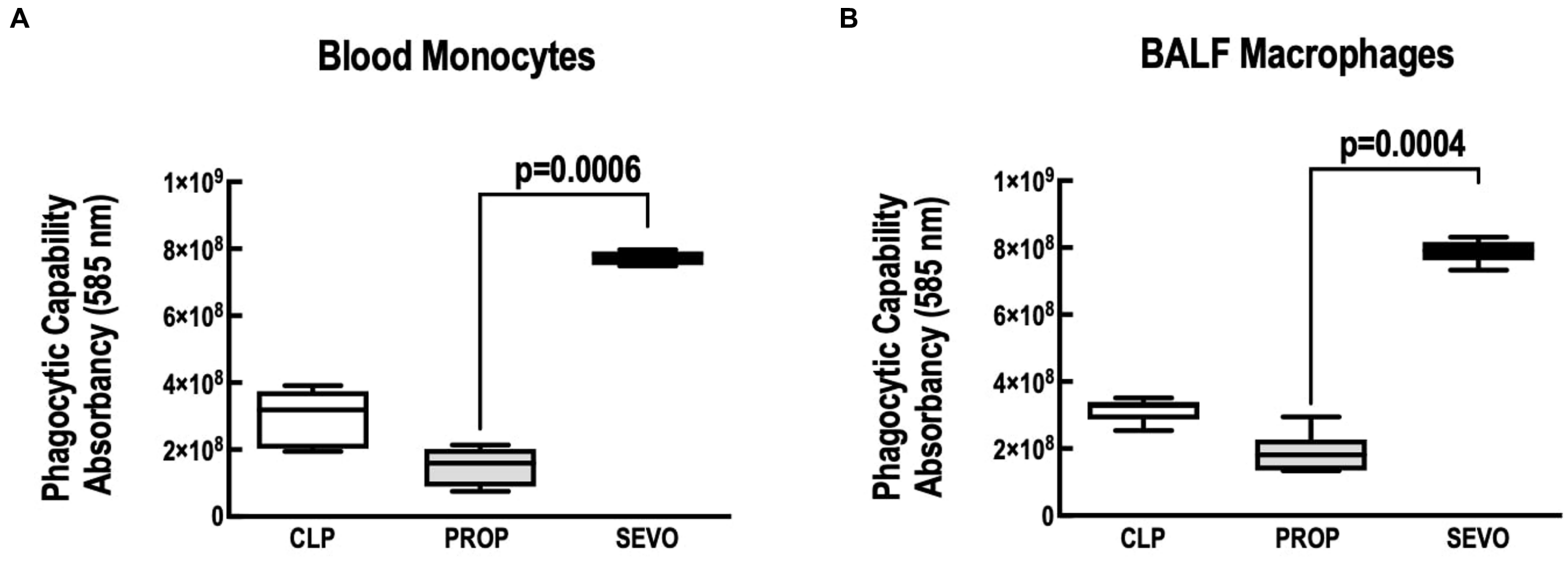
Phagocytosis assay. **(A)** Blood monocytes. **(B)** BALF macrophages. BALF, bronchoalveolar lavage fluid; CLP, cecum ligation and puncture, control group, not exposed to anesthetics; MAC, minimum alveolar concentration; PROP, propofol group, exposed to 50 μM propofol; SEVO, sevoflurane group, exposed to sevoflurane 1 MAC. Boxes show the interquartile (25%–75%) range, whiskers encompass the range (minimum to maximum), and horizontal lines represent median values of 9 animals/group.

The phagocytic capacity of blood monocytes (Figure 3A) and BALF macrophages (Figure 3B) (*p* = 0.0006, *p* = 0.004, respectively) was higher with sevoflurane compared with propofol. No differences were observed between CLP versus PROP and CLP versus SEVO.

**Figure 4:**
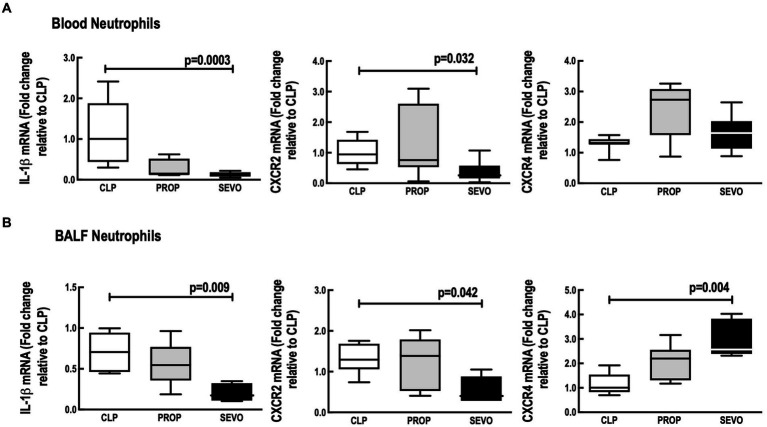
mRNA of mediators expressed by blood **(A)** and BALF **(B)** neutrophils. BALF, bronchoalveolar lavage fluid; CLP, cecum ligation and puncture, control group, not exposed to anesthetics; MAC, minimum alveolar concentration; PROP, propofol group, exposed to 50 μM propofol; SEVO, sevoflurane group, exposed to sevoflurane 1 MAC; IL, interleukin; CXCR, C-X-C motif chemokine receptor. Boxes show the interquartile (25%–75%) range, whiskers encompass the range (minimum to maximum), and horizontal lines represent median values of 9 animals/group.

In neutrophils from blood (Figure 4A) and BALF (Figure 4B), compared with CLP, sevoflurane exposure, reduced mRNA expression of IL1-β (*p* = 0.0003, *p* = 0.009, respectively), and CXCR2 (*p* = 0.032, *p* = 0.042, respectively). Sevoflurane also increased mRNA expression of CXCR4 (*p* = 0.004, Figure 4B) in BALF neutrophils, but no significant differences were observed in blood neutrophils (Figure 4A). No differences were observed in blood or BALF neutrophils mRNA expression of IL1-β, CXCR2 and CXCR4 between CLP versus PROP and PROP versus SEVO.

**Figure 5:**
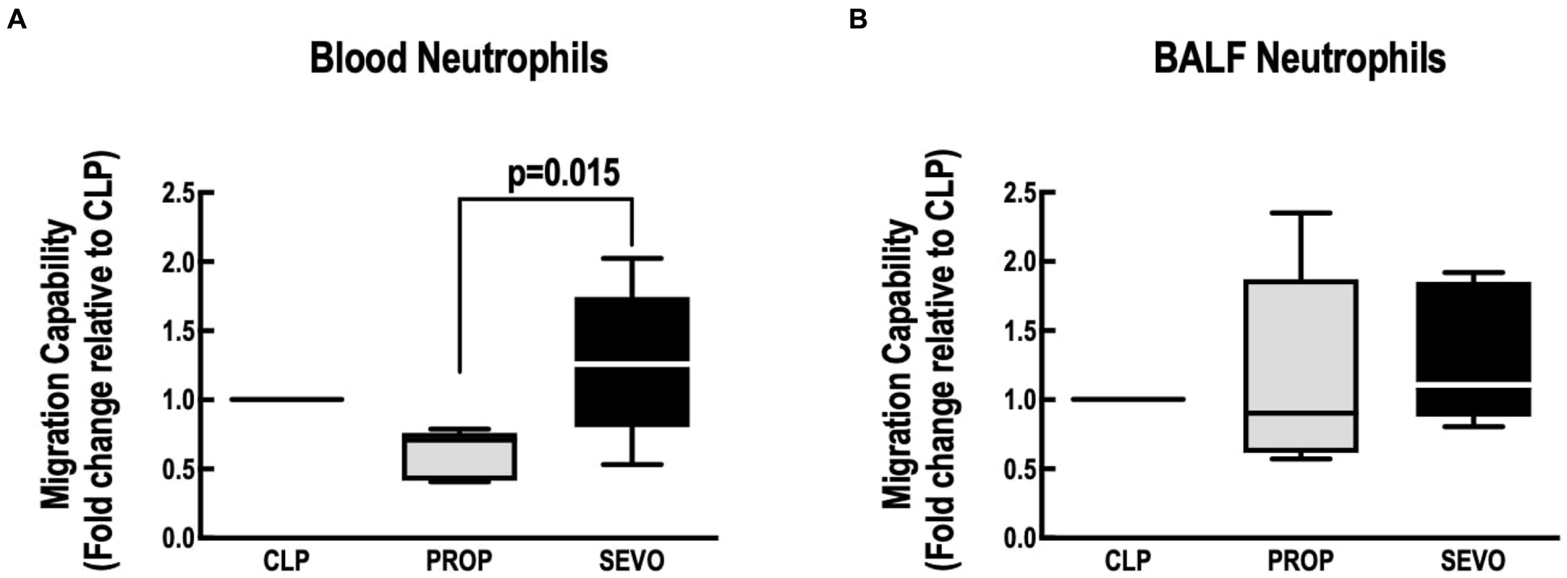
Neutrophil migration assay. Migration capacity of blood neutrophils **(A)** and BALF neutrophils **(B)**. BALF, bronchoalveolar lavage fluid; CLP, cecum ligation and puncture, control group, not exposed to anesthetics; MAC, minimum alveolar concentration; PROP, propofol group, exposed to 50 μM propofol; SEVO, sevoflurane group, exposed to sevoflurane 1 MAC. Boxes show the interquartile (25%–75%) range, whiskers encompass the range (minimum to maximum), and horizontal lines represent median values of 9 animals/group.

Compared with propofol, Sevoflurane increased neutrophil migration in blood (*p* = 0.015, Figure 5A) but not in the BALF (Figure 5B). No differences were observed between CLP versus PROP, and CLP versus SEVO in blood or BALF neutrophils.

**Figure 6:**
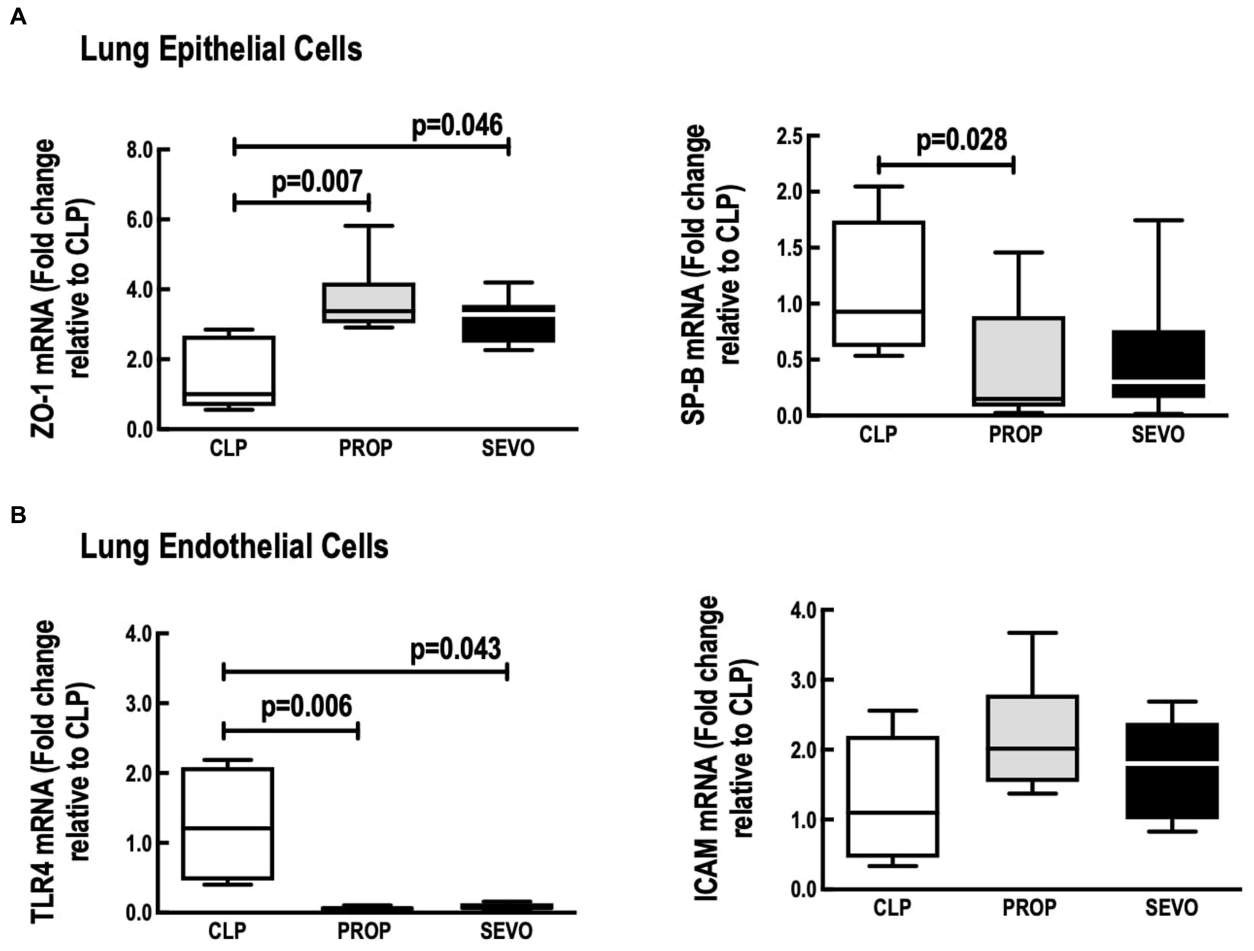
Lung structural cells. mRNA expression of **(A)** epithelial cells, **(B)** endothelial cells. CLP, cecum ligation and puncture, control group, not exposed to anesthetics; MAC, minimum alveolar concentration; PROP, propofol group, exposed to 50 μM propofol; SEVO, sevoflurane group, exposed to sevoflurane 1 MAC; SP-B, surfactant protein B; TLR4, Toll-like receptor 4; ZO-1, zona occludens 1. Boxes show the interquartile (25%–75%) range, whiskers encompass the range (minimum to maximum), and horizontal lines represent median values of 9 animals/group.

In alveolar epithelial cells, compared to CLP, sevoflurane and propofol exposure increased mRNA ZO-1 (*p* = 0.046, *p* = 0.007, respectively) and only propofol reduced SP-B mRNA expression (*p* = 0.028, Figure 6A). No differences were observed in ZO-1 and SP-B between PROP and SEVO groups.

In lung endothelial cells (Figure 6B), TLR4 mRNA expression was reduced regardless of the anesthetic agent (*p* = 0.043, SEVO; *p* = 0.006) compared to CLP, whereas no difference was observed between PROP and SEVO groups. ICAM-1 mRNA did not differ among all the groups.

Main results were summarized in graphical abstract.

## 4. Discussion

In this study, cells obtained from septic animals respond differently to 1-h exposure to propofol and sevoflurane (see graphical abstract). No previous studies have compared the impact of sevoflurane and propofol on blood monocytes/BALF macrophages, neutrophils, or on structures such as alveolar epithelial and endothelial cells, which are important in the pathophysiology of sepsis. Polymicrobial sepsis induced by CLP is widely used because of its established protocol (21) and close resemblance to the progression and features of human sepsis. We improved the CLP protocol by adding antibiotic treatment after induction of sepsis to better mimic clinical practice, as suggested by a consensus initiative on improving animal modeling in sepsis (36). We evaluated sevoflurane and propofol because they are widely used in perioperative medicine (4, 11) and have immunomodulatory activity, lung and extra-pulmonary protective effects in critical illness (10, 13). Apart from *in vivo* studies, it is difficult to evaluate the effects of a given anesthetic agent in specific cells at different compartments during sepsis. Circulating and resident cells at the alveolar compartment were obtained, primarily cultured, and then exposed to clinical concentrations of two widely used anesthetic agents for 1 h. Sevoflurane (at a similar dosage as in our study, 1.2 MAC) improved survival in a murine CLP sepsis model (16). We observed that mRNA of inflammatory biomarkers (IL-6 and IL-1β) were reduced in isolated blood monocytes and neutrophils after exposure to sevoflurane for 1 h. This is in line with a previous report showing that sevoflurane can decrease NF-κB translocation into the nucleus by inducing both protein [inhibitor of nuclear factor kappa B (IκB)] stabilization and upregulation in a human monocytic cell line, THP-1 cells (17). Moreover, blood monocytes exposure to sevoflurane, but not to propofol, exhibited increased mRNA expression of anti-inflammatory mediators, TGF-β and IL-10. During the early phase of sepsis, restauration of immunosuppressive cytokines, such as IL-10, may be in line with organ function recovery and improved survival (16).

Blood monocytes and BALF macrophages exposure to sevoflurane resulted in higher phagocytic compared to propofol. Modulation of macrophage phagocytic capacity after volatile anesthetic is controversial (18, 37, 38). Phagocytic capacity of monocytes from patients undergoing cardiac catheterization who received volatile anesthetics did not change (37). In contrast, sevoflurane reduced macrophage phagocytosis *in vitro* using RAW264.7 cells, thioglycollate-induced mouse peritoneal macrophages, and phorbol myristate acetate-stimulated THP-1 cells (38). The immortalized cells (RAW264.7, THP-1) and artificial stimuli (thioglycollate and phorbol myristate acetate), although used at the bench, do not trigger the same response observed during sepsis or regular infection (39). In a previous endotoxemia model, sevoflurane enhanced macrophage phagocytosis *in vitro* through an inducible nitric oxide synthase (iNOS)-dependent mechanism (18). During sepsis, pro-inflammatory cytokines such as TNF-α and IL-1β are released by sentinel innate immune cells at the infection site and activated by pathogen-associated molecular pattern (PAMPS) and damage-associated molecular pattern (DAMPS) (40). These cytokines activate neutrophils, lead to increased expression of β-integrins on the cell surface of neutrophils, and interact with highly expressed adhesion molecules on the vascular endothelium and neutrophils leave the vasculature based on chemoattractant gradient (41). We found that exposure to sevoflurane for 1 h reduced mRNA expression of IL-1β in blood and BALF neutrophils. In parallel, sevoflurane reduced mRNA expression of CXCR2 in both sources of neutrophils. Among other functions, CXCR2 signaling activates the NF-κB pathway, inducing transcription of many cytokines, including CXC chemokines that amplify neutrophil recruitment, and is associated with tissue damage (42). Reduction of CXCR2 might explain the better performance of sevoflurane in attenuating expression of neutrophil inflammatory genes. CXCR2 signaling is a chemokine pathway that interacts antagonistically with CXCR4 to regulate neutrophil release from bone marrow. CXCR2 induces recruitment of bone marrow neutrophils into blood circulation, and CXCR4 induces cell retention in bone marrow (43). Circulating neutrophils express low levels of CXCR4, which is upregulated in senescent neutrophils before apoptosis, promoting homing back to the bone marrow and other organs for clearance (43). Increased expression of CXCR4 was observed in blood neutrophils after exposure to propofol and in BALF neutrophils after sevoflurane. In the early phase of sepsis, characterized by an exacerbated pro-inflammatory response, sevoflurane might induce better results because it can impair recruitment from bone marrow neutrophils by reducing CXCR2 and inducing clearance from neutrophils from the alveolar compartment, which are associated with poor outcomes in acute respiratory distress syndrome induced by sepsis. Despite reducing production of inflammatory mediators, neither sevoflurane nor propofol had an impact on neutrophil migration to the cytokine gradient, which is important, because the ability of neutrophils to respond to sites of infection during the acute phase of sepsis has been shown to be impaired (44).

During the early phase of sepsis, pulmonary edema can manifest by increasing alveolar epithelial permeability. This is depicted, among other factors, by reduced expression of tight junction proteins between alveolar epithelial cells. Among them, ZO-1 is the bridge connecting occludin and the cytoskeleton. Changes in occludin and ZO-1 expression are closely related to pulmonary tissue permeability (45). In our study, we observed that ZO-1 mRNA was upregulated after exposure to sevoflurane and propofol. Sevoflurane may keep the integrity of the alveolar-capillary barrier through the modulation of ZO-1 expression in alveolar epithelial cells (45). Although no previous studies have evaluated the effect of propofol on ZO-1 gene expression in alveolar epithelial cells during sepsis, it has been shown that propofol has the ability to increase the levels of tight junctions proteins, such as occludin, in the blood–brain barrier after hypoxia (46). SP-B, a hydrophobic protein, is considered the most important protein for sustaining respiratory physiology. SP-B levels increase during lung infection and disease resolution is associated with restoration of the levels of SP-B (47). Only propofol reduced SP-B mRNA expression in alveolar epithelial cells in agreement with a previous *in vivo* study in an endotoxin-induced acute lung injury model in rats (48), in which the administration of propofol was associated with a decrease of SP-B mRNA in the lungs, and animals exposed to sevoflurane presented higher mRNA expression of SP-B, better lung oxygenation and reduced lung injury, compared to propofol (48). Finally, Sevoflurane and propofol reduced TLR4 gene expression in lung endothelial cells, likely due to decreased NF-κB activation (6, 17).

In this study, we have isolated epithelial and endothelial cells and kept them in culture until confluence, that, in average, took 1 week. We acknowledge that cultured endothelial/epithelial cells cannot recapitulate all *in situ* endothelial/epithelial cells features, because of cell plasticity when removed from their environment (49). Nonetheless, in previous studies from our group, lung endothelial and epithelial cells isolated from CLP-induced sepsis model (24) were kept in culture until confluence, similarly to experiments run in this study. CLP-derived cells showed increased oxidative stress and reduced mitochondrial respiration compared to SHAM animals. Thus, from our experience, 1 week from isolation under culture conditions, endothelial and epithelial cells still keep some features from the cells residing in diseased lungs (24) and can be used as an *ex vivo* platform for drug studies, with translational value (49).

It has been demonstrated that, at clinical concentrations, different anesthetics depress the functions of the inflammatory response differentially (50, 51). Besides sevoflurane and propofol, we have used midazolam and ketamine to sedate and anesthetize the animals for CLP surgery. Benzodiazepines, such as midazolam, are known to inhibit certain aspects of immune function, but midazolam infusion did not affect cytokine production in septic patients (52). The immune-inhibitory effects of ketamine were recently found to be partly due to inhibition of transcription factor activator protein-1 and nuclear factor-kappa B (NF-κB), which can regulate the production of several proinflammatory mediators (53). Thiopental induce LPS-stimulated mononuclear cells to produce anti-inflammatory cytokines such as IL-10 (54). We have used these drugs, and we acknowledge they might act in the immune system. However, midazolam’s terminal half-life is 3.1 h (55), ketamine’s is1h (56), and animals were all animals were submitted to them 48 h before the tested anesthetics (sevoflurane and propofol). Despite thiopental’s half-life is 5.89 h (57), it was used for euthanasia in all animals, and the likely inflammation attenuation induced by other pharmacological agents was homogeneously distributed among the groups.

Short exposure times have been used to study anesthetic immunological properties (58, 59). Exposure of 1 h (59) or 2 h (58) of intravenous and volatile anesthetics have been tested *in vitro* and showed immunosuppressive effects. The effects of repetitive anesthesia with sevoflurane has also been studied, and showed that three short exposures to the anesthetic (weekly, 40 min each) induce persistent humoral response alterations (60). In parallel, the occurrence of major postoperative infectious complications in patients undergoing esophagectomy was best predicted by increased duration of anesthesia, and not by surgical procedure or operation time, showing the importance of anesthesia exposure time (61). In our study, we have showed that the exposure time of 1 h can be enough for identifying cellular and molecular pathways but may not show every detail regarding their physiological consequences in longer periods.

The present study has some limitations. First, this *in vitro* model has limitations mostly inherent to the translational value of the study, especially in relation to the challenge in faithfully reproducing the complex pathophysiology of sepsis. Although we used polymicrobial sepsis induced by CLP model with adaptations to better replicate the clinical scenario of sepsis, it is still not possible to guarantee higher levels of fidelity with those found in *in vivo* and clinical models. Second, we used only male rats in our model. We know that sex steroids synergistically modulate immune and cardiovascular responses in infectious diseases and sepsis. Females have been shown to be protected under these conditions, whereas males can be vulnerable due to decreased cell-mediated immune response and cardiovascular functions (62). Therefore, our study, using only male Wistar rats, evaluated the immunomodulatory action of anesthetics in a scenario of greater severity of sepsis, because male sex is associated with reduced cellular immune response. Third, despite the biomarkers measured on lung epithelial and endothelial cells may not provide functional information, we studied the neutrophils migration, which can infer cell adhesion into structural cells. Fourth, according to our research question, we aimed to study the anesthetic exposure on inflammatory and structural cells in a separate way. Although, it seems a substantial isolation process, it took around 50 min to perform the experiments for inflammatory cells and 7 days, for structural cells. This period of time has been shown to not change cell’s features (24). Fifth, this study presents data that evaluate the mRNA expression of the genes of choice, which cannot necessarily be interpreted in a similar way to protein quantification. Sixth, we did not include cells from control animals, once it would increase the number of groups and reduce the statistical power of the present study.

This manuscript describes a proof-of-concept study aiming to identify the impact of both anesthetics in an *in vitro* CLP-induced polymicrobial sepsis model. We plan to further evaluate the impact of anesthetics in an *in vivo* study, focusing not only in inflammatory or structural cells, but in lung function and mortality. In our study, we opted to first identify potential cells that could be influenced by sevoflurane and propofol in order to better guide what could be evaluated in *in vivo* condition. There are some pre-clinical studies evaluating the effect of the anesthetics tested herein in models of sepsis or acute respiratory distress syndrome models (16, 63, 64). Sevoflurane has been able to reduce mortality in CLP-model of sepsis (16). When compared to propofol, sevoflurane was able to reduce systemic inflammation, but not neuro-inflammation in a LPS-inflammation model (63), and, ameliorates lung inflammatory response and improves oxygenation to a greater extent than propofol in ARDS model (64) In parallel, their immunomodulatory effects have been also compared in other different clinical scenarios. In patients undergoing flap breast reconstruction, patients anesthetized with propofol showed less increase in syndecan-1 level, an endothelial glycocalyx injury marker, then patients that received sevoflurane (65). In patients undergoing craniotomy, patients that received propofol had higher levels of the anti-inflammatory IL-10 compared to patients that received sevoflurane (66). The literature on the immunomodulatory effects of anesthetics is still scarce and somewhat controversial. Thus, besides hemodynamic and neurological impact, anesthesists should also take into consideration anesthetics have immunomodulation properties, and their superiority might vary according to clinical condition.

## 5. Conclusion

Sevoflurane, compared with propofol, increased anti-inflammatory biomarkers in monocytes, but not in alveolar macrophages, enhanced monocyte/alveolar macrophage phagocytic capacity and increased neutrophil migration in *in vitro* experimental sepsis. Both propofol and sevoflurane protected lung epithelial and endothelial cells.

Personalized selection of sedative and/or anesthetic agents may affect the pathophysiology of sepsis and be critical in the clinical management of these patients.

## Data availability statement

The original contributions presented in the study are included in the article/supplementary material, further inquiries can be directed to the corresponding author.

## Ethics statement

The animal study was reviewed and approved by Institutional Animal Care and Use Committee of the Health Sciences Centre, Federal University of Rio de Janeiro (CEUA 027/17).

## Author contributions

TO, DB, PP, PR, PS, and FC worked in the design and conduct of the study. TO, CB, and FC worked in data collection and analysis. TO, FC, and PS have written the manuscript. All authors contributed to the article and approved the submitted version.

## Funding

This work was supported by the Brazilian Council for Scientific and Technological Development (CNPq), the Rio de Janeiro State Research Foundation (FAPERJ), the Department of Science and Technology (DECIT)/Brazilian Ministry of Health, and the Coordination for the Improvement of Higher Education Personnel (CAPES).

## Conflict of interest

The authors declare that the research was conducted in the absence of any commercial or financial relationships that could be construed as a potential conflict of interest.

## Publisher’s note

All claims expressed in this article are solely those of the authors and do not necessarily represent those of their affiliated organizations, or those of the publisher, the editors and the reviewers. Any product that may be evaluated in this article, or claim that may be made by its manufacturer, is not guaranteed or endorsed by the publisher.
